# Remote sensing technology for rapid extraction of burned areas and ecosystem environmental assessment

**DOI:** 10.7717/peerj.14557

**Published:** 2023-02-06

**Authors:** Shiqi Zhang, Maoyang Bai, Xiao Wang, Xuefeng Peng, Ailin Chen, Peihao Peng

**Affiliations:** 1 College of Earth Sciences, Chengdu University of Technology, Chengdu, China; 2 School of Architecture and Civil Engineering, Chengdu University, Chengdu, China; 3 College of Tourism and Urban-Rural Planning, Chengdu University of Technology, Chengdu, China; 4 Sichuan Earthquake Agency, Chengdu, China; 5 Chengdu lnstitute of Tibetan Plateau Earthquake Research, China Earthquake Administration, Chengdu, China

**Keywords:** Forest fire, Burned areas, Sentinel-2, Remote sensing environment index, GEE platform, OTSU threshold

## Abstract

Forest fires are one of the significant disturbances in forest ecosystems. It is essential to extract burned areas rapidly and accurately to formulate forest restoration strategies and plan restoration plans. In this work, we constructed decision trees and used a combination of differential normalized burn ratio (dNBR) index and OTSU threshold method to extract the heavily and mildly burned areas. The applicability of this method was evaluated with three fires in Muli County, Sichuan, China, and we concluded that the extraction accuracy of this method could reach 97.69% and 96.37% for small area forest fires, while the extraction accuracy was lower for large area fires, only 89.32%. In addition, the remote sensing environment index (RSEI) was used to evaluate the ecological environment changes. It analyzed the change of the RSEI level through the transition matrix, and all three fires showed that the changes in RSEI were stronger for heavily burned areas than for mildly burned areas, after the forest fire the ecological environment (RSEI) was reduced from good to moderate. These results realized the quantitative evaluation and dynamic evaluation of the ecological environment condition, providing an essential basis for the restoration, decision making and management of the affected forests.

## Introduction

Forests, the terrestrial ecosystems’ mainstay, are the foundation of human survival and development (Mitchard, 2018; Gao et al., 2020). Forest fires are fast-spreading, sudden and difficult to control. They cause massive forest damage and severely affect the composition of ecosystems, leading to a dramatic reduction in biomass and drastic changes in the landscape and the global carbon cycle (Xaud, da Martins & Dos Santos, 2013; De Faria et al., 2017; Liu, Ballantyne & Cooper, 2019; Zhao et al., 2021b, 2021a). In recent years, global warming and frequent human production activities have increased forest fires worldwide (Hall et al., 2016; Forkel et al., 2019; Vargas-Cuentas & Roman-Gonzalez, 2021). The forest fire prevention industry is facing a more complex situation, so new requirements and challenges for the restoration work after the disaster (Andela et al., 2017). It is critical to rapidly and accurately extract the extent of burned areas in forests, enabling the timely development of forest restoration strategies and plans (Pezzatti et al., 2020; Kim, Hwang & Choi, 2021). Traditionally, the burned area of forest fires is delineated by manual field surveys, but this method is generally labor-intensive and costly because of the complex topography of the southwestern forest area (Mitri & Gitas, 2008; Gitas et al., 2012). Satellite remote sensing has significant advantages in extracting the burned areas and assessing ecological changes in the forest fires, with its short imaging period and wide monitoring range. The theory is that vegetation reduces chlorophyll content after fire burning, and the reduction of chlorophyll causes wavelength changes in the visible, red-edge and near-infrared (NIR) wavelengths (Mueller et al., 2017; Ba et al., 2019; Reddy et al., 2019; Chuvieco et al., 2020; Laneve et al., 2020; Santana et al., 2020; Sudhakar et al., 2020). The index threshold method is the most commonly used, which separates the burned area by analyzing the spectral reflectance curve of the burned area, selecting the appropriate waveband to establish the index, and choosing the appropriate threshold to separate the burned area. The Burned Area Index (BAI) (Chuvieco, Martín & Palacios, 2010), Normalized Burn Ratio (NBR) (Cocke, Fulé & Crouse, 2005), Mid-InfraRed Bispectral Index (MIRBI) (Smith et al., 2007) and other indices were derived successively. In addition, the Normalized Divergence Vegetation Index (NDVI), Soil-Adjusted Vegetation Index (SAVI), Global Environment Monitoring Index (GEMI), Enhanced Vegetation Index (EVI) and other remote sensing vegetation indices are also commonly used in burned areas mapping (Sun et al., 2019). It has been found that NBR can be used as an indicator of burn severity in forested areas (Epting, Verbyla & Sorbel, 2005). Several global burned areas products have been released in the past few years, mainly from remote sensing satellite sensors such as MODIS, MERIS, or VEGETA, which have a higher temporal resolution but a lower spatial resolution (>300 m) and cannot extract burned area more accurately (Hall et al., 2016; Forkel et al., 2019; Santana et al., 2020; Valencia et al., 2020; Franquesa et al., 2022). Sentinel-2 and Landsat data, which can provide more detailed high-resolution remote sensing images, also bring further accuracy improvement to the regional forest burned area extraction (Roteta et al., 2021). Traditional threshold determination is an artificially selected threshold, which may artificially bias the results (Giri, Qiu & Zhang, 2017; Sekertekin, 2021). The OTSU algorithm is a classical adaptive thresholding segmentation algorithm, and it uses the maximum inter-class variance adaptation to determine the optimal threshold to achieve the best segmentation effect. The OTSU algorithm is computationally simple and independent of image brightness and contrast, so it is widely used in automated extraction (Otsu, 1996; Huang & Jin, 2020; Pan, Xi & Wang, 2020; Dutta, Talukdar & Bora, 2022). Fire can cause forest damage to different degrees, generally divided into fire-traced areas (called mildly burned areas) and forest loss areas (called heavily burned areas) (Rao, Wang & Huang, 2020). Available burned area products do not break down the burned areas with different damage (Santana et al., 2020; Valencia et al., 2020). However, the length of restoration and subsequent management of different damaged forests is different, so it is necessary to classify the burned areas. Decision Tree Learning (DTL) is a critical remote sensing extraction method in the process of statistics, data mining and machine learning, which divides samples through hierarchical progressive attribute judgments. The decision tree is widely used in remote sensing image classification extraction as an efficient yet simple machine learning method (Yang et al., 2003; Zhou, Zhang & Zhang, 2016; Verma, Garg & Hari Prasad, 2017; Zhang et al., 2017; Ghatkar, Singh & Shanmugam, 2019). There have been studies using the decision tree to build a combination of the burning index and OTSU thresholds to extract burned areas (Tianchi, Fuqin & Yan, 2020; Li et al., 2021a; Tang et al., 2021; Tian et al., 2022), which can achieve better accuracy results. There are also comparative studies on the extraction of the burned area from Sentinel-2 and Landsat 8 data sources, and they found that Sentinel generally performed as well or better than Landsat for four spectral indices of burn severity and that Sentinel’s higher spatial resolution improves opportunities for examining finer-scale fire effects across ecosystems (Mallinis, Mitsopoulos & Chrysafi, 2018; Zhang et al., 2019; Howe et al., 2022). However, the extraction and evaluation of burned areas with different damage using Sentinel-2 are lacking, so assessing the method’s applicability is necessary. The Google Earth engine (GEE) is a tool that can batch-process satellite image data. It can process large amounts of images quickly and in batches compared with traditional tools for processing images, and is one of the most popular remote sensing processing tools available. It is also starting to be used in burned area extraction (Roteta et al., 2021; Seydi et al., 2021). In this study, we evaluated the applicability of the dNBR and OTSU threshold methods based on the GEE platform for the extraction of heavy and mildly burned areas from three forest fires.

On the other hand, the ecological environment of burned areas changes significantly after the fire, such as soil, moisture, and biology. Firstly, the structure and quantity of plants and leaves are limited, with a corresponding decrease in chlorophyll and overall environmental moisture. The most apparent change is the increase in soil temperature, the magnitude of which is closely related to fire intensity, fire duration, and soil moisture content. These effects can be reflected by moisture, greenness, heat and dryness indicators: soil moisture and vegetation water content caused changes in wetness. Vegetation health status caused changes in greenness, soil temperature and ground cover caused changes in heat, soil temperature and land degradation caused dryness changes (Attorre et al., 2015; Chia et al., 2016; Day et al., 2020). There are some studies on the dynamic changes of vegetation in the study area before and after forest fires (Zhou et al., 2019), but less monitoring of ecological damage before and after the forest fires. Remote sensing technology has strong advantages for monitoring ecological changes. The remote sensing ecological index (RESI) is entirely based on remote sensing information technology, which reduces the subjectivity and difficulty of index extraction in practical applications and facilitates large-scale and continuous investigation and observation (Xu, 2013). It can be used to reflect the comprehensive status of regional ecological quality (Jiang et al., 2021; Yuan et al., 2021; Lian et al., 2022; Wang & Li, 2022). It couples four indicators that intuitively reflect the superiority or inferiority of ecological quality status, such as wetness, greenness, heat and dryness, characterized by humidity index, normalized vegetation index, surface temperature and dryness index, respectively.

Muli County is located in the Southwest Forest Region, one of the most frequent areas where forest fires occur in China. Muli County had forest fires for three consecutive years in 2019, 2020 and 2021. It has a complex landscape with high and low relief and rugged closed terrain. Once a fire occurs, the high mountain and steep slope environment accelerates the fire’s spread and exacerbates the difficulty of extinguishing it. Therefore, Muli County is a high-risk area for forest fires and a key area of national fire risk (Li et al., 2021b). In this work, taking Muli County, where fires occurred for three consecutive years, as the study area, we used the dNBR index of sentinel-2 remote sensing images, combined with the OTSU thresholding method and decision tree to extract the heavily burned areas and mildly burned areas. In addition, we evaluated the ecological environment changes by using RSEI and analyzing them to achieve the quantitative evaluation of ecological environment status and dynamic evaluation.

## Materials and Methods

**Figure 1:**
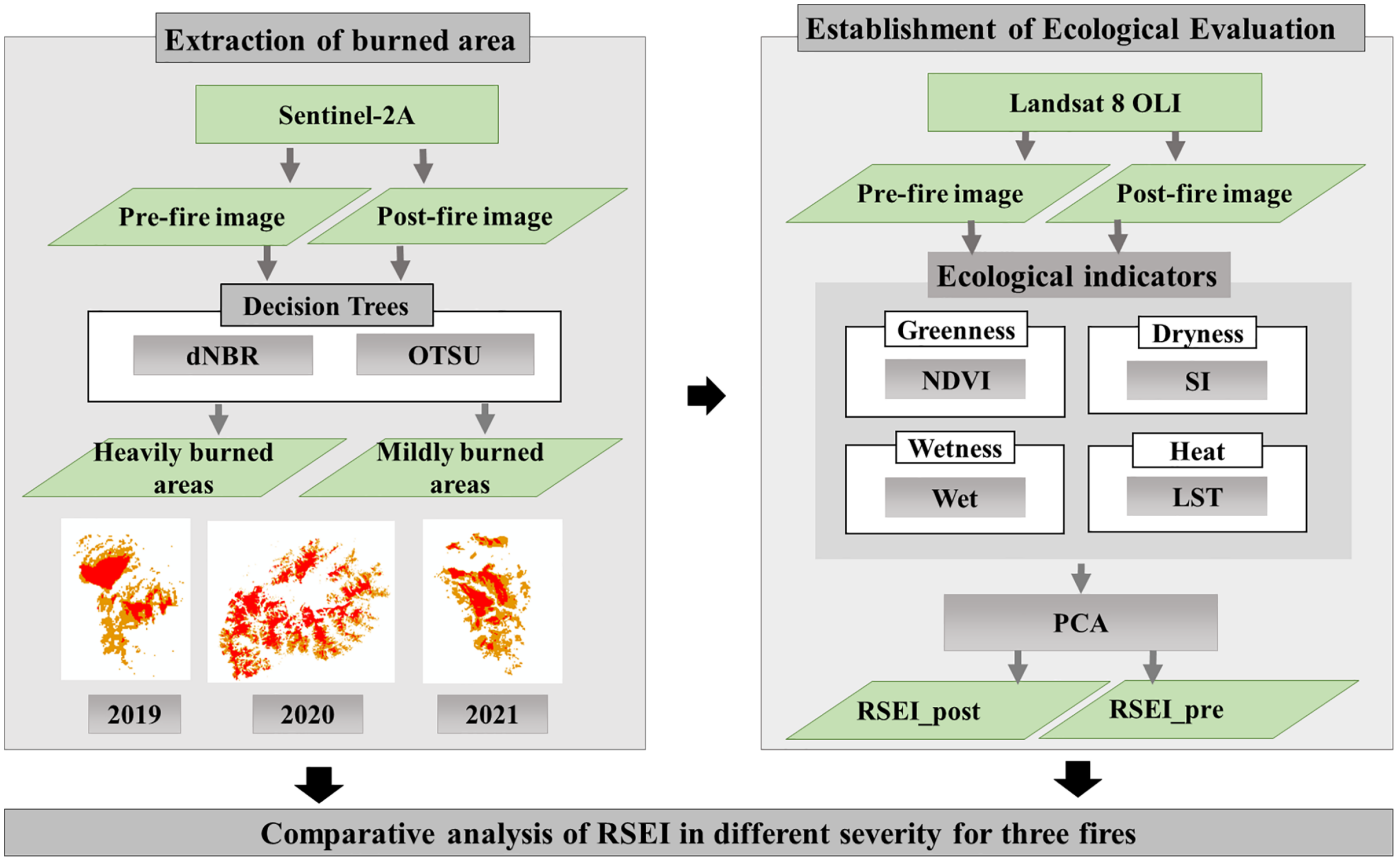
The workflow.

This work is divided into three main sections. The materials and methods are shown in the following Figure (Fig. 1).

Extraction of the burned area

This work used the GEE cloud platform to rapidly extract the burned area caused by three major fires in Muli County in 2019, 2020, and 2021, and the extraction process is as follows: Firstly, Sentinel-2A images were acquired one month after the fire was extinguished, and the median was synthesized as the post-fire image; Considering the phenological cycle of the vegetation in the study area, the Sentinel-2A images were also acquired one year before that period, and the median was synthesized as the pre-fire image, and the specific image acquisition time is shown in the following Table 1.

**Table 1:** Time of the images acquisition.

Time of fire	Post-fire image	Pre-fire image
March 30, 2019	April 10–May 10, 2019	April 10–May 10, 2018
March 28, 2020	April 7–May 7, 2020	April 7–May 7, 2019
April 5, 2021	April 9, 2021–May 9, 2021	April 9, 2021–May 9, 2020

Secondly, we constructed a decision tree to extract the burned area; we used the NIR and SWIR bands to calculate the NBR index in pre-fire images and the NBR index in post-fire images, and calculated the dNBR, then the OTSU algorithm was used to calculate the dNBR threshold1 to extract heavily burned area, and used this result to mask the heavily burned area and keep the other parts. Finally, the OTSU algorithm was used again to classify and extract the mildly burned areas.

Establishment of ecological evaluation

The remote sensing ecological index (RSEI) be used to evaluate the ecological and environmental of the burned area. It integrates moisture, greenness, heat and dryness information using principal component analysis.

Comparative analysis of RSEI in different severity for three fires

We resample the extracted burning area to 30 m, with the same spatial resolution of RSEI, and compare the variation of RSEI for different severity for three fires.

### Study area

Muli County is located on the southeastern edge of the Qinghai-Tibet Plateau (longitude 100°03′–101°41′ east, latitude 27°40′–29°10′ north), at the boundary between the Qinghai-Tibet Plateau and the Yunnan-Guizhou Plateau. Its main landform features are mountains, canyons and mountain plains. The terrain is complex, and the altitude difference a are enormous. It belongs to a typical alpine and canyon area. Muli County has a subtropical monsoon climate, with rainy summers and less rain in winter. Muli is rich in forest resources, with a forest coverage rate of 67.3%, including *Picea asperata*, *Quercus semicarpifolia*, *Pinus densata*, *etc*., and more than 10 kinds of national first and second-class protected tree species. The volume of standing trees is 117 million m^2^, accounting for 10% of Sichuan Province and 1% of the whole country (Liu, 2014), and the vegetation is vertically distributed.

**Figure 2:**
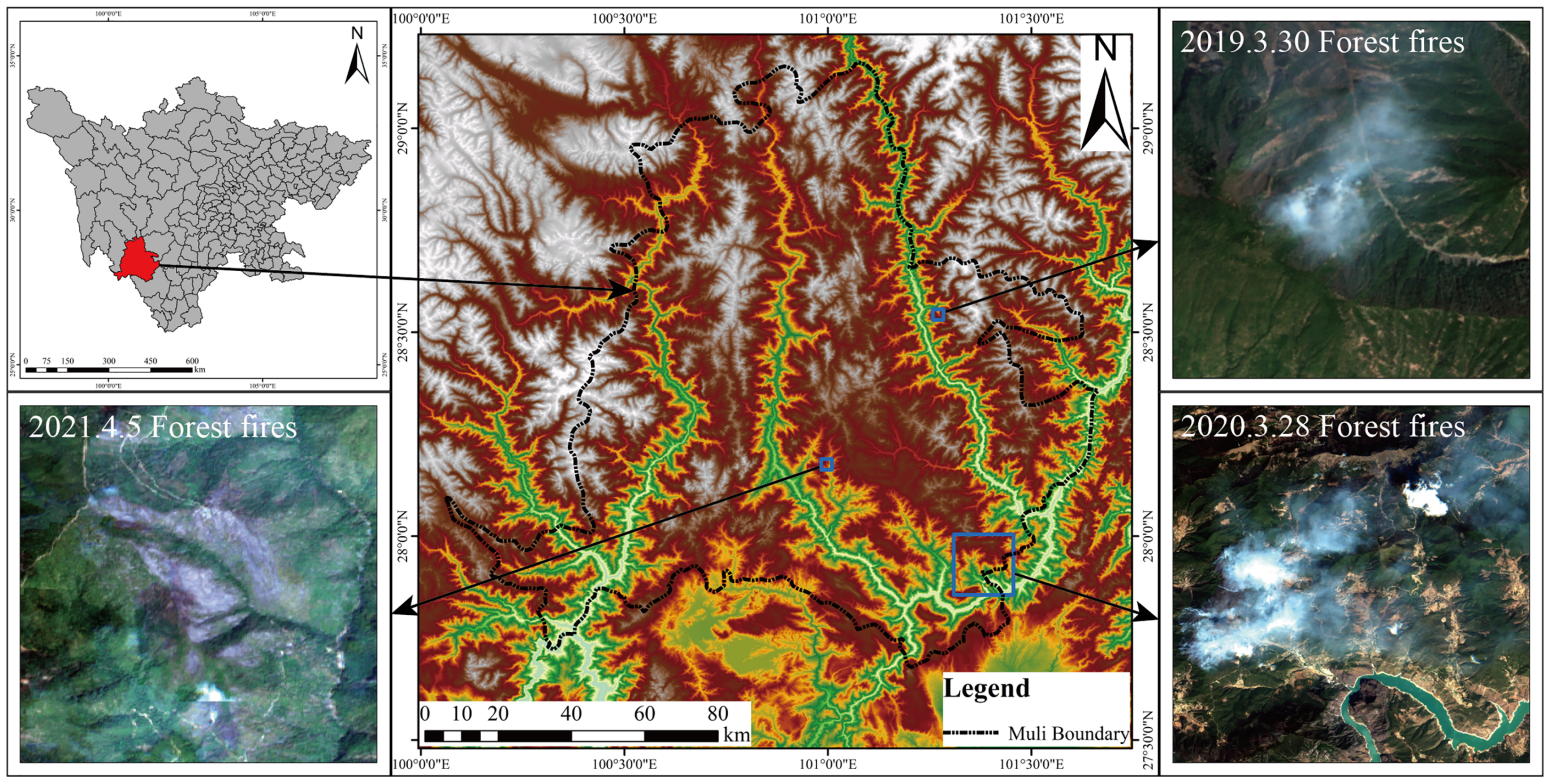
Location of the study area (forest fires images are from Sentinel-2).

The annual average temperature is about 14.0 °C, the temperature difference between day and night is significant, and solar radiation is powerful. The four seasons are not pronounced, and the dry and wet seasons are distinct. The dry season is from December to March of the following year., the climate is dry, with little precipitation, long sunshine hours, and uneven spatial and temporal distribution of precipitation, often suffering from natural disasters such as droughts, debris flows and floods (Li et al., 2021d, 2021c). The wet season is from June to September, the precipitation is concentrated, the rainfall is abundant, the daily temperature difference is slight, and the climate is suitable. The forest in the study area is widely distributed, the terrain is complex and changeable, and the forest fire prevention situation is complicated. Forest fires have occurred in the region for three consecutive years, causing massive casualties, economic losses and loss of forest resources. The fire areas are shown in Fig. 2:

### Data

#### Satellite images

The remote sensing images used in this work are sentinel-2 and Landsat 8 OLI from the Google Earth Engine Cloud Platform (https://code.earthengine.google.com/). The Sentinel-2 L2A dataset, which ESA had pre-processed for radiometric calibration, atmospheric correction, *etc*., reflects the reflectance information at the surface; this dataset is used to extract the burned areas in the study area. The Landsat 8 Level 2 Collection 2 Tier 1 is a reflectance product created by the Land Surface Reflectance Code (LaSRC) and processed by a single-pass algorithm, including radiometric and geometric correction datasets. Both datasets can be used without subsequent processing. The data information is shown in Table 2:

**Table 2:** Data information.

Datasets	Temporal resolution	Spatial resolution
Sentinel-2 L2A	5 days	10 m
Landsat 8 Level 2 Collection 2 Tier 1	16 days	30 m

### Reference data

The reference data are the fire survey data of the Sichuan Forestry Survey and Design Institute. It was vector data that was visually interpreted from sentinel-2 remote sensing images and corrected using field verification. The reference data mainly includes the forest loss area (heavily burned area), the forest where fire passed area (forest loss is not apparent, mildly burned area), and various survey data details are shown in Table 3.

**Table 3:** The statistical table of fire area survey.

Time of fire	Burned area (hm^2^)		
	Heavily burned area	mildly burned area	Total burned area
March 30, 2019	43.90	74.19	118.09
March 28, 2020	8,856.05	8,547.84	17,403.89
April 5, 2021	66.12	150.26	216.38

### dNBR computation

The NBR (Normalized Burn Ratio) is a remote sensing index obtained by replacing the red band in the NDVI (Normalized Difference Vegetation Index) equation with the SWIR band. Since the reflectance change in the NIR in the burned areas is more significant than that in the SWIR compared with the non-burned areas, it can be used as an essential indicator factor to extract burned areas. The NBR values range from 1 to −1 and negatively correlate with forest fire intensity. However, the NBR index has some advantages in the extraction of burned areas, but using the mono-temporal NBR is still affected by low reflectivity areas such as water bodies, which is prone to significant errors. The dNBR (delta Normalized Bun Ratio) (Chen, Loboda & Hall, 2020) utilized a multi-temporal that is the difference of NBR index in the post-fire images and pre-fire images, and it can effectively reduce the influence of factors such as clouds and aerosols compared with the NBR index.

Moreover, it can be seen that the NBR reflectance of burned areas showed an increase after the occurrence of forest fires. In contrast, the SWIR reflectance of burned areas is extremely sensitive to water bodies, and the vegetation humidity in the forest decreases after burning, so the higher the intensity of the forest fire, the more significant the decrease in humidity within the forest and the decrease in humidity then leads to the decrease the SWIR reflectance. Finally, the NBR index shows a decrease. Therefore, the dNBR index of the burned area images is significantly higher than the low reflectance areas such as water bodies. In summary, the dNBR index of multi-temporal can be used to distinguish the burned areas. The NBR’s and the dNBR’s mathematical formulas are as Eqs. (1) and (2):

(1) $$NBR = \displaystyle{{\rho {}_{nir} - {\rho _{swir}}} \over {\rho {}_{nir} + {\rho _{swir}}}}$$

(2) $$dNBR = NB{R_{pre}} - NB{R_{post}}$$

where ${\rho _{nir}}$ represents the NIR band reflectance of sentinel-2 (Band 8); ${\rho _{swir}}$ represents the SWIR band reflectance of sentinel-2 (Band 11); $NB{R_{pre}}$ represents the pre-fire NBR index; $NB{R_{post}}$ represents the post-fire NBR index.

### OTSU thresholding for image segmentation

Threshold segmentation is one of the most commonly used image segmentation methods; it is simple and effective. Moreover, scholars have made researched the methods of automatically obtaining thresholds in image segmentation work. The OTSU algorithm, also known as the maximum interclass variance method, is an automatic algorithm for the global binarization of clustered images proposed by Japanese scholar Zhenyuki Otsu in 1979 (Otsu, 1979). It can obtain more ideal segmentation results, has the characteristics of high segmentation efficiency and broad application scenarios, the results are stable, and the segmentation quality can be guaranteed, so it has been widely used (Fan et al., 2016). It works as a discrete simulation of the one-dimensional Fisher discriminant. In computer vision and image processing work, this algorithm assumes that an image contains only two classes of pixels (background pixels and target pixels) after bimodal histogram processing and then minimizes their intra-class variance and maximizes inter-class variance by calculating the best threshold that can distinguish between the two classes of pixels.

The OTSU algorithm is computed as follows: Assuming that an image has several pixels, the probability of occurrence in each gray-level pixel ${P_i}$ is Eq. (3):

(3) $${P_i} = {\raise0.7ex\hbox{${{n_i}}$} \!\mathord{\left/ {\vphantom {{{n_i}} N}}\right.} \!\lower0.7ex\hbox{$N$}},(i = 0,1,2,3\ldots L - 1),\sum\nolimits_{i = 0}^{L - 1} {{P_i} = 1}$$

where the grayscale $i$ takes a range $\left[ {0,L - 1} \right]$, the number of pixels in the grayscale $i$ level is ${n_i}$. The image was divided into background pixels ${C_0}$ and target pixels ${C_1}$ using thresholding, ${C_0}$ consisting of pixels with grayscale values $\left[ {0,k} \right]$ and ${C_1}$ pixels with grayscale values $\left[ {t + 1,L - 1} \right]$. Then, the probability of occurrence of each gray ${\mu _t}$ is Eq. (4):

(4) $${\mu _t} = \sum\nolimits_{i = 0}^{L - 1} {i{P_i}}$$

The occurrence probability of ${C_0}$ and ${C_1}$ pixels are Eqs. (5) and (6):

(5) $${\omega _0} = \sum\nolimits_{i = 0}^{L - 1} {{P_i}}$$

(6) $${\omega _1} = \sum\nolimits_{i = t + 1}^{L - 1} {{P_i}} = 1 - {\omega _0}$$

The average gray ${\mu _0}$ and ${\mu _i}$ are Eqs. (7)–(9):

(7) $${\mu _0} = \sum\nolimits_{i = 0}^t {{\raise0.7ex\hbox{${i{P_i}}$} \!\mathord{\left/ {\vphantom {{i{P_i}} {{\omega _0}}}}\right.} \!\lower0.7ex\hbox{${{\omega _0}}$}}}$$

(8) $${\mu _1} = \sum\nolimits_{i = t + 1}^{L - 1} {{\raise0.7ex\hbox{${i{P_i}}$} \!\mathord{\left/ {\vphantom {{i{P_i}} {{\omega _1}}}}\right.} \!\lower0.7ex\hbox{${{\omega _1}}$}}}$$

(9) $${\mu _t} = {\omega _0}{\mu _0} + {\omega _1}{\mu _1}$$

The interclass variance $\delta _t^2$ is Eq. (10):

(10) $$\delta _t^2 = {\omega _0}{({\mu _0} - {\mu _t})^2} + {\omega _1}{({\mu _1} - {\mu _t})^2} = {\omega _0}{\omega _1}{({\mu _0} - {\mu _1})^2}$$

The optimal threshold to distinguish between the two classes of pixels is the one that takes the value in the interval when the maximum value is reached.

After a forest fire, there is a sharp increase in the maximum interclass variance within a reasonable image element window, and the OTSU algorithm was applied to extract the dNBR threshold automatically. It can be used as an essential parameter in active fire detection.

### Building decision trees

Decision tree learning is an essential method in the field of statistics, data mining and machine learning, mainly used as a prediction model to analyze test and target variables in a round-robin manner and finally to predict the category of the samples. The training process is to obtain rules for classification by summarizing expert experience, simple statistical analysis of mathematical models, induction, *etc*. A training set is divided into several subsets, and the recursion is repeated in the resulting subsets. This top-down decision tree induction method is one of the greedy algorithms and is one of the most commonly used training methods, which can be applied in predicting continuous variables and remote sensing classification.

**Figure 3:**
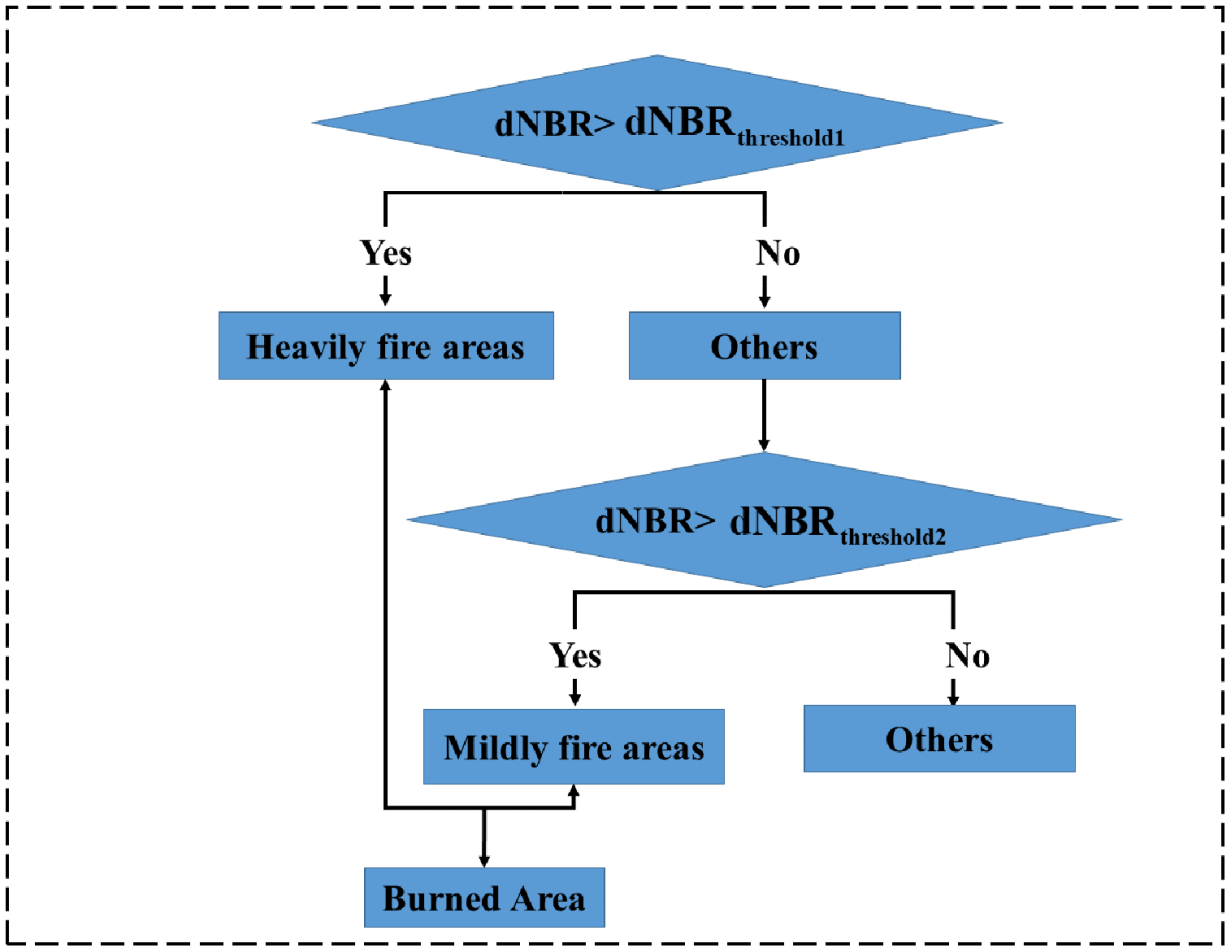
Decision tree classification model.

In this work, we constructed a decision tree classification model with the dNBR based on the sensitive feature analysis of burned areas. First, we divided burned areas into the mildly burned areas (fire traced area) and the heavily burned area (forest loss area) and calculated the first dNBR threshold based on OTSU, which was used to distinguish the heavily burned area (forest loss area). Then a second dNBR threshold is calculated in the rest area based on OTSU, which is used to distinguish between the mildly burned areas (fire traced area) as well as the rest area, and the decision tree classification model is shown in Fig. 3.

### Accuracy verification

The accuracy verification of burned areas identification used the following calculation formula (Eq. (11)):

(11) $${\rm P} = (1) - \left| {\displaystyle{{{\rm a} - {\rm b}} \over {\rm b}}} \right|\times 100\%$$

*P* represents the accuracy of the method, *a* represents the number of image elements of the identified burning area, and *b* represents the number of image elements visually interpreted after the field survey respectively.

To better verify the experimental results, we used the kappa coefficient to verify the accuracy. Its equation is as follows.

(12) $${\rm Kappa} = \displaystyle{{{{\rm p}_{\rm o}} - {{\rm p}_{\rm e}}} \over {1 - {{\rm P}_{\rm e}}}}$$

where ${{\rm P}_{\rm o}}$ is the sum of the diagonals of the confusion matrix divided by the total number of samples, and ${{\rm P}_{\rm e}}$ is the sum of the “product of actual and predicted numbers” for each of the categories, divided by the “square of the total number of samples”.

### Construction of a comprehensive ecological index

Therefore, the remote sensing ecological index (RSEI) is rapid monitoring and evaluation of the ecological method based on natural factors, which integrates moisture, greenness, heat and dryness information and can also be used to evaluate the ecological and environment of the burned area.

### Greenness

In this work, the normalized vegetation index (NDVI) was selected to characterize the greenness of the study area, which can reflect the plant biomass, leaf area index and vegetation cover in the region, calculated as Eq. (13):

(13) $$NDVI = \displaystyle{{{\rho _{nir}} - {\rho _{red}}} \over {{\rho _{nir}} + {\rho _{red}}}}$$

where, ${\rho _{nir}}$ and ${\rho _{red}}$ represent the reflectance of Landsat images in the NIR and red bands, respectively.

### Wetness

The wetness index is characterized based on the moisture component obtained from the tassel cap transformation, and the wetness can reflect the surface water body conditions, especially the moisture of the soil and the calculation method for wetness extraction based on Landsat8/OLI data is shown in Eq. (13) (Baig et al., 2014). Before extraction, the water bodies were masked using the Modified Normalized Difference Water Index (MNDWI) (Sun, Wu & Tan, 2012), so the wetness reflects the actual land surface moisture conditions.

(14) $${\rm Wet} = 0.1511{{\rm b}_2} + 0.1973{{\rm b}_3} + 0.3283{{\rm b}_4} + 0.3407{{\rm b}_5} - 0.7117{{\rm b}_6} - 0.4559{{\rm b}_7}$$

where: ${{\rm b}_2},\, {{\rm b}_3},\, {{\rm b}_4},\, {{\rm b}_5},\, {{\rm b}_6},\, {{\rm b}_7}$ represents the reflectance of Landsat8/OLI images in bands 2, 3, 4, 5, 6, and 7, respectively; Wet represents the humidity component of Landsat8/OLI images.

### Heat

This work used Landsat 8 surface temperature to invert the land surface temperature (LST) to indicate the hotness indicator. The dual-band feature of Landsat 8 thermal infrared data offers the possibility of using a split-window algorithm to invert the surface temperature. Therefore, single-channel surface temperature inversion methods are mainly used in surface temperature inversion work to estimate surface temperature. This article chose the single window algorithm (SMW) proposed by Ermida et al. (2020). The SMW algorithm is based on an empirical relationship between TOA brightness temperatures in a single TIR channel and LST and utilizes simple linear regression. The model consists of a linearization of the radiative transfer equation that maintains an explicit dependence on surface emissivity, and it is calculated as Eq. (15).

(15) $$LST = {A_i}\displaystyle{{{T_b}} \over \varepsilon } + {B_i}\displaystyle{1 \over \varepsilon } + {C_i}$$

where ${T_b}$ is the TOA brightness temperature in the TIR channel, and $\varepsilon$ is the surface emissivity for the same channel. The algorithm coefficients ${A_i}$; ${B_i}$; and ${C_i}$ are determined from linear regressions of radiative transfer simulations performed for 10 classes of Total Column Water Vapor (TCWV) that values from NCEP/NCAR reanalysis data are available on GEE (I = 1, …, 10), ranging from 0 to 6 cm in steps of 0.6 cm, with values of TCWV above 6 cm being assigned to the last class.

(4) Dryness

The dryness index represents an indicator of land dryness, which also indicates the degree of soil dryness and the land is affected by weathering and sanding. The dryness index consists of the normalized building bare soil index (NDBSI) incorporating the bare soil index (SI) and the building index (IBI), and the burned areas are mainly affected by the SI, and most burned areas are far from the towns and not easily affected by the IBI. Rikimaru’s model was used to calculate the following Eq. (16).

(16) $$SI = \displaystyle{{({\rho _{swir1}} + {\rho _{red}}) - ({\rho _{blue}} + {\rho _{NIR}})} \over {({\rho _{swir1}} + {\rho _{red}}) + ({\rho _{blue}} + {\rho _{NIR}})}}$$

where, ${\rho _{blue}},\, {\rho _{red}},\, {\rho _{swir1}},\, {\rho _{NIR}}$ denotes the reflectance of the blue band, red band, near-infrared band, and short-wave infrared band1 corresponding to the OLI image, respectively.

The proposed ecological index should be able to appear both as a single indicator and to combine the information from the above four indicators. Therefore, we used principal component analysis (PCA) (Gewers et al., 2021), a multivariate statistical method, to construct the ecological index. It is a multi-dimensional data compression technique that can select a few critical variables by an orthogonal linear transformation. Its most significant advantage is that the weights of the integrated indicators are not determined artificially; instead, they are determined automatically and objectively according to the contribution of each indicator to each principal component, thus avoiding the bias of the results caused by the different weight settings from a person.

Since the four indices are not uniform, it is necessary to normalize the original values of the four indices to a range between [0, 1] to avoid the imbalance of each index weight in the principal component analysis, and the normalization formula is as Eq. (17):

(17) $$BI = {{({I_i} - {I_{min}})} \mathord{\left/ {\vphantom {{({I_i} - {I_{min}})} {({I_{max}} - {I_{min}})}}} \right.} {({I_{max}} - {I_{min}})}}$$

where, *BI* is the normalized image element value of a factor, $I{}_i$ is the image element value of a factor, $I{}_{max}$ and $I{}_{min}$ the maximum and minimum values of the factor, respectively, the normalized indexes have a scale between 0 and 1. The normalized metrics are combined into a new image, and a principal component analysis is performed on the new image to obtain the first principal component (PC1) and related statistics. The obtained PCl is normalized to obtain the RSEI.

## Results

### The extraction results of burned areas

According to the OTSU calculation, the heavily burned threshold and mildly burned threshold for each fire was obtained and then can get the heavily burned areas and mildly burned areas, and the two areas were added to get the total burned area. The results are shown in the following Table 4.

**Table 4:** Extraction results of burned areas and their accuracy.

Time of fire	Heavy fire				Mild fire				Total burned area	
	dNBR_threshold1	Burned area (hm^2^)	Accuracy	Kappa	dNBR_ threshold2	Burned area (hm^2^)	Accuracy	kappa	Burned area (hm^2^)	Accuracy
March 30, 2019	0.3010	43.20	98.4%	0.87	0.0654	77.62	95.38%	0.74	120.82	97.74%
March 28, 2020	0.3789	9,574.57	91.89%	0.81	0.1211	9,680.25	86.75%	0.71	19,254.82	89.32%
April 5, 2021	0.4024	66.81	98.96%	0.84	0.1464	159.59	93.79%	0.69	226.40	96.37%

From Table 4, we can see that the accuracy of burned area extracted by combining the dNBR and OTSU thresholding image segmentation method for three fires was 97.69%, 89.32%, and 96.37%, respectively. The method performs better in the accuracy of small burned areas, but the performance is average in the extraction of the large burned area; the accuracy of heavily burned area extraction is higher than that of mildly burned.

**Figure 4:**
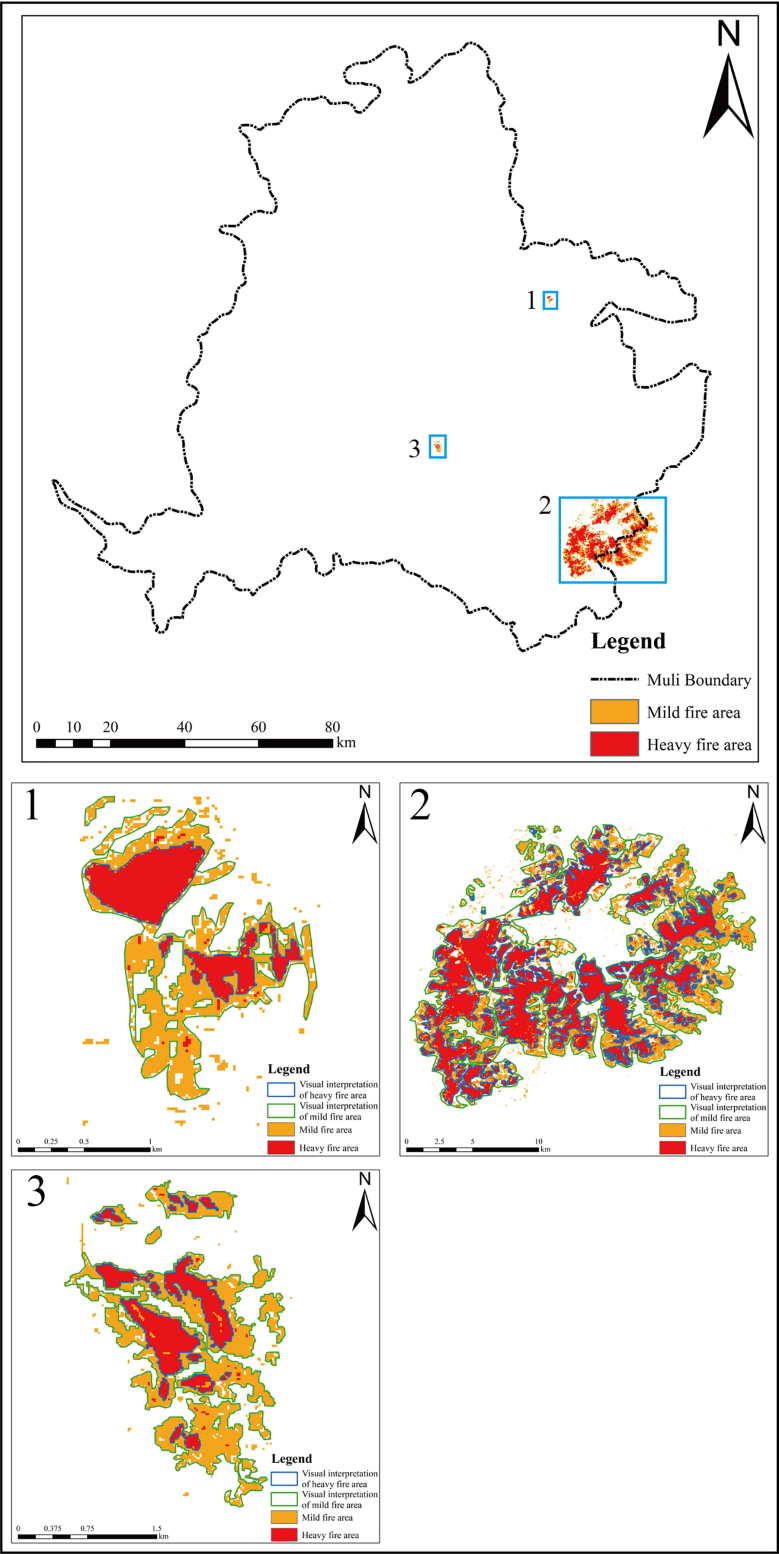
The burned area extraction map.

Combined with the actual visual interpretation areas, the burned areas map derived from GEE using the OTSU threshold image segmentation method was compared, as shown in Fig. 4.

As can be seen, the heavily burned areas that were extracted matched the visual interpretation area, and the proposed method’s accuracy is 98.4%, 91.89% and 98.96%, respectively. In contrast, the mildly burned areas are mainly distributed near the heavily burned areas, and the extracted burned areas are complete with clear boundaries and close to the visual interpretation results. However, it is difficult to intuitively extract the mildly burned areas and discriminate them from the remote sensing images.

### Ecological environment evaluation results

As can be seen from Table 5, NDVI and wetness both played a positive role in promoting the ecological environment of the study area during the period, while SI and LST played a negative role together, which is consistent with the actual situation. The PC1’s contribution in the calculation of the RSEI was more outstanding than 90%, indicating that the PC1 had concentrated most of the four indicators’ information in the three fires; meanwhile, the four component indicators’ contribution to PC1 was relatively stable, so PC1 was chosen to create the RSEI instead of the four component indicators.

**Table 5:** The results of PCA.

		PC1	PC2	PC3	PC4
2019	Wet	0.6099	0.4007	0.3483	0.5884
	NDVI	0.5874	0.3296	0.2112	0.7083
	LST	−0.4084	−0.4847	−0.7028	−0.3228
	SI	−0.3409	−0.7042	−0.5831	−0.2189
	%	94.62	3.81	1.22	0.35
2020	Wet	0.6809	0.5059	0.3991	0.3480
	NDVI	0.4609	0.2654	0.6608	0.5296
	LST	−0.3879	−0.5829	−0.4239	−0.5745
	SI	−0.4165	−0.5778	−0.4735	−0.5181
	%	93.9	4.71	1.01	0.39
2021	Wet	0.6692	0.3997	0.6130	0.1289
	NDVI	0.4924	0.2753	0.5972	0.5702
	LST	−0.4878	−0.3993	−0.4107	−0.6587
	SI	−0.2677	−0.7778	−0.3145	−0.4737
	%	97.64	1.54	0.61	0.21

The RESI’s mean values were calculated from the results of PCA in before and after the fire as shown in the following Table 6.

**Table 6:** RSEI’s mean values.

RSEI_mean_	2019		2020		2021	
	Heavy fire area	Mild fire area	Heavy fire area	Mild fire area	Heavy fire area	Mild fire area
Pre-RSEI	0.6623	0.6674	0.6953	0.6971	0.7270	0.7484
Post-RSEI	0.4928	0.6036	0.5321	0.5910	0.4239	0.5894
Difference	0.1695	0.0638	0.1632	0.1061	0.3031	0.4106

**Figure 5:**
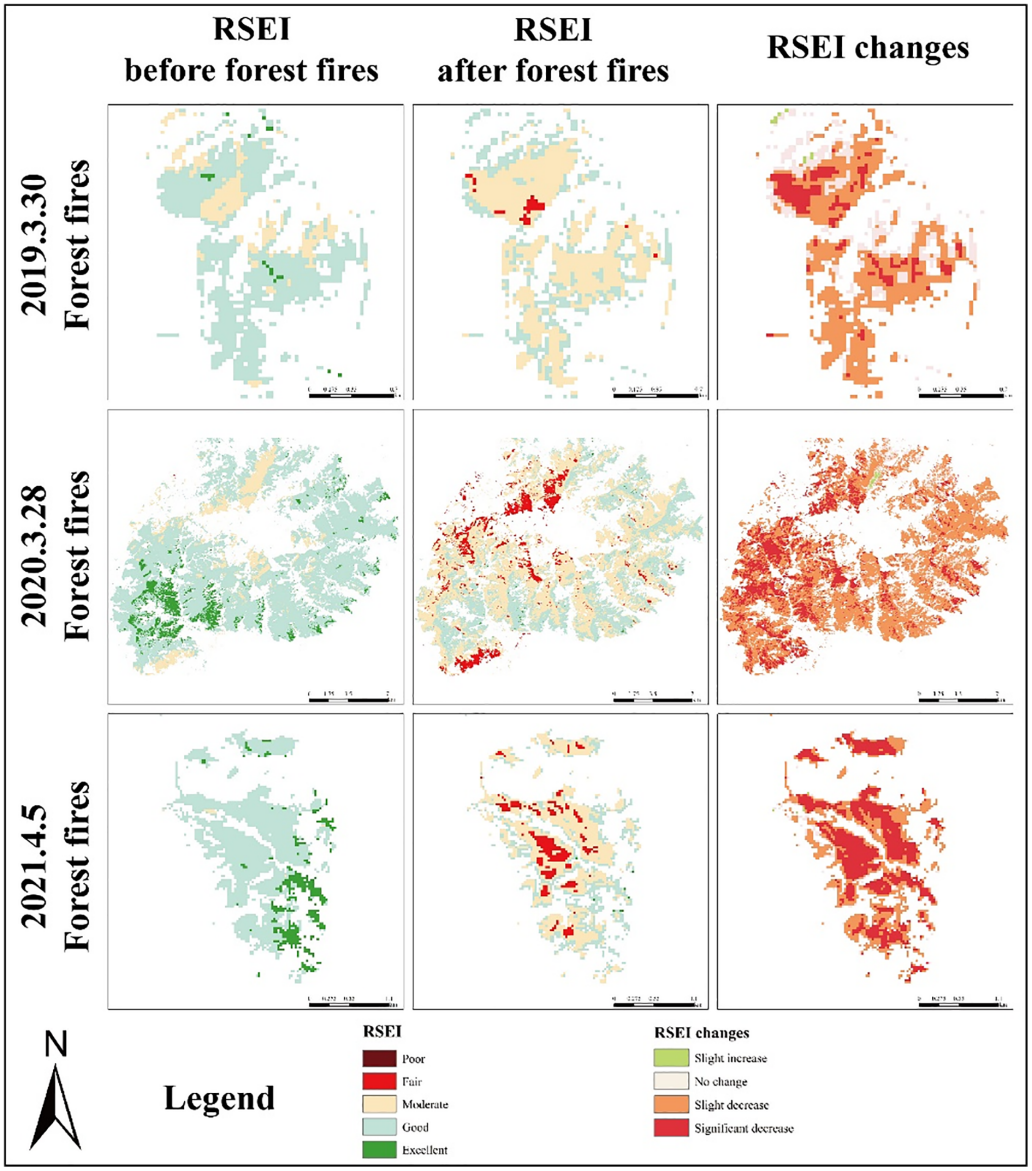
RSEI changes map.

We have concluded that the RSEI decreases significantly after three fires. To finely analyze the changes of RSEI, we calculated the difference of RESI before and after the fire for each pixel. Where −0.2 to −0.05 is a slight increase, −0.05 to 0.05 is no significant change, 0.05–0.20 is a slight decrease, and greater than 0.20 is a significant decrease. As shown in Fig. 5 below:

As we can see the Fig. 5, the following phenomena were present in all three fires: The post-RSEI of mildly burned and heavily burned was significantly lower than that of pre-RSEI, indicating that the fires brought different degrees of damage to the ecological environment; meanwhile, the difference in RSEI of heavy burned was higher than that of mildly burned, indicating that the damage of RSEI in the area of heavy burned was more serious, and the vegetation restoration measures in the area should be considered to reduce the impact of burned on the ecological environment as early as possible.

### The monitoring analysis of the ecological environment

To further investigate the changes in the local ecological conditions, the normalized RSEI ecological index was divided into five levels according to the 0.2 value interval, from low to high: excellent: 0.8–1.0; good: 0.6–0.8; moderate: 0.4–0.6; fair: 0.2–0.4; poor: 0–0.2. We calculated the area and proportion of ecological classes before and after the three fires. And the transfer matrix was used to analyze the ecological changes as follows.

In 2019, we can see (Table 7) that the ecological environment of the burned areas changed: after the burn, the ecological environment had no excellent, but the poor ecological environment had an area of 0.09 hm^2^; In the mildly burned area, the ecological environment changed from good to medium with an area of 34.26 hm^2^, the changing area accounted for 49.8%, and all the excellent ecological environment changed to good. The ecological environment of the heavily burned area had changed more, and it has a poor with an area of 2.59 hm^2^, the excellent ecological environment (1.08 hm^2^) changed to a medium (0.99 hm^2^) ecological environment with accounted for 95.12%, which means that the ecological environment of the heavily burned area has changed more prominently.

**Table 7:** RSEI’s transfer matrix for heavily burned (a) and mildly burned (b) in 2019.

		Fire_pre					
		Poor	Fair	Moderate	Good	Excellent	Total
(a)							
Fire_post	Poor	0	0	0	0	0	0
	Fair	0	0	0.09	0	0	0.09
	Moderate	0	0	8.02	34.26	0	42.28
	Good	0	0	0	34.53	0.72	35.25
	Excellent	0	0	0	0	0	0
	Total	0	0	8.11	68.79	0.72	
	Change	0	0	0.09	34.26	0.72	
(b)							
Fire_post	Poor	0	0	0	0	0	0
	Fair	0	0	1.51	1.08	0	2.59
	Moderate	0	0	7.81	30.12	0.99	38.92
	Good	0	0	0	1.6	0.09	1.69
	Excellent	0	0	0	0	0	0
	Total	0	0	9.32	32.8	1.08	
	Change	0	0	1.51	31.2	1.08	

In 2020, there was no poor ecological environment before the fire (Table 8), but poor ecological conditions appeared after the fire with an area of 13.88 hm^2^. The areas of the excellent ecological environment also decreased from 1,370.5 to 57.06 hm^2^. In the mildly burned areas, the ecological environment was concentrated in the good ecological environment before the fire, but after the fire, its ecological environment changed to medium grade, accounting for 49.73% (3,962.3 hm^2^), and the ecological environment changed from excellent to good grade, and it accounted for 92.30%. In the heavily burned area, the ecological environment evaluation was concentrated in the good grade, but it was concentrated in the medium grade after the fire. Changes in the ecological environment were concentrated in the pre-fire good and excellent ecological environment, and most of the excellent ecological environment was changed to the medium or poor ecological environment, with accounted for 98.90%, and most of the good ecological environment was changed to the medium ecological environment with accounted for 72.90%.

**Table 8:** RSEI’s transfer matrix for heavily burned (a) and mildly burned (b) in 2020.

		Fire_pre					
		Poor	Fair	Moderate	Good	Excellent	Total
(a)							
Fire_post	Poor	0	0.54	2.88	0.72	0.09	4.23
	Fair	0	0.72	310.08	122.67	3.06	436.53
	Moderate	0	0	513.99	3,962.3	70.74	4,547.03
	Good	0	0	0	4,130.75	512.75	4,643.5
	Excellent	0	0	0	0	48.96	48.96
	Total	0	1.26	826.95	8,216.44	635.6	
	Change	0	0.54	312.96	4,085.69	586.64	
(b)							
Fire_post	Poor	0	0	4.45	5.11	0.09	9.65
	Fair	0	0.09	539.52	516.75	7.55	1,063.91
	Moderate	0	0	476.81	5,178.1	202.12	5,857.03
	Good	0	0	0	2,118.84	517.04	2,635.88
	Excellent	0	0	0	0	8.1	8.1
	Total	0	0.09	1,020.78	7,818.8	734.9	
	Change	0	0	543.97	5,699.96	726.8	

Also, we can see (Table 9) that the ecological environment of the burned area changed in 2021: before the fire, there was no poor ecological environment, but after the fire, there was a poor ecological environment with an area of 27.38 hm^2^, while the excellent ecological environment changed from 32.68 to 0.63 hm^2^ with accounted for 61.51%, and the excellent ecological environment changed to good or medium with accounted for 97.93%; In the heavily burned area: medium ecological environment all changed to the worse ecological environment with accounted for 100%, and good ecological environment changed to the worse or medium ecological environment with accounted for 99.02%, excellent ecological environment mainly changed to the medium and worse ecological environment with accounted for 100%.

**Table 9:** RSEI’s transfer matrix for heavily burned (a) and mildly burned (b) in 2021.

		Fire_pre					
		Poor	Fair	Moderate	Good	Excellent	Total
(a)							
Fire_post	Poor	0	0	0	0	0	0
	Fair	0	0	0.09	25.85	0.09	26.03
	Moderate	0	0	0	38.01	1.51	39.52
	Good	0	0	0	0.63	0.63	1.26
	Excellent	0	0	0	0	0	0
	Total	0	0	0.09	64.49	2.23	
	Change	0	0	0.09	63.86	2.23	
(b)							
Fire_post	Poor	0	0	0	0	0	0
	Fair	0	0	0.09	1.26	0	1.35
	Moderate	0	0	0.9	78.83	10.7	90.43
	Good	0	0	0	48.06	19.12	67.18
	Excellent	0	0	0	0	0.63	0.63
	Total	0	0	0.99	128.15	30.45	
	Change	0	0	0.09	80.09	29.82	

In summary, there was no poor ecological environment in the three pre-fires, but the ecology showed a poor level after being burned in high fire intensity in 2020 and no poor level after the 2019 and 2021 fires. So in terms of area burned and ecological changes, the fires in 2020 were more severe than those in 2019 and 2020. On the other hand, regardless of the heavily burned area and mildly burned area, the pre-fire ecology had a good area, but its post-fire ecology change area was also the largest, and the post-fire ecology had the most moderate area. It shows that the ecological environment changed from good to moderate after the fire.

## Discussion

Forest fires are frequent and costly, so it is crucial to use remote sensing techniques to quickly extract burned areas and assess their ecological change (Liu, Ballantyne & Cooper, 2019; Mota et al., 2019). Remote sensing images have the advantages of low cost, high accuracy, and high efficiency, and they play an essential role in fire scene identification and burning.

Remote sensing burn indices are often used for extracting burned areas, but most of the studies have focused on major fires (burned area over 100 km^2^) (Tian et al., 2022), and the applicability of the method has rarely been evaluated. On the other hand, the often used remote sensing data, such as sentinel-2, Gaofen, *etc*., have a high spatial resolution, large storage space and low data processing efficiency, which means that their wide industrial application may be limited. In order to get accurate information and dynamics of forest fires, efficient and reliable forest fire identification algorithms are especially important. In this study, using sentinel-2 remote sensing data on the GEE platform, we used the idea of a decision tree to extract the burning area of three fires in Muli County by combining the dNBR and OTSU thresholding methods. Accuracy comparing the results of the method using the remote sensing technology with the published research results.

Among them, in 2019, compared with Rao, Wang & Huang (2020), the accuracy of heavy burning was improved by 3.73%, the accuracy of light burning was improved by 4.44%, and the overall accuracy was improved by 7.03%. In 2020, Li et al. (2021a) extracted a burned area of 180.37 km^2^ by calculating the Difference Normalized Vegetation Index (dNDVI) from Landsat 8 remote sensing imagery km. However, the area visually interpreted in that study was 204.54 km^2^, while the area extracted in this study was 192.55 km^2^, while Tian et al. (2022), based on the visual interpretation results of Li et al. extracted the burning area of 209.17 km^2^ using Sentinel-2 images combined with dNBR, which improved the accuracy compared with the area of 204.54 km^2^ based on the visual interpretation of Landsat 8 remote sensing images. However, the empirical threshold method was used in that study. In this study, the burned area extracted by using the OTSU automatic extraction threshold method in this study is 192.55 km^2^, which is close to the fire survey data of the Sichuan Forestry Survey and Design Institute obtained visual interpretation based on Sentinel-2 remote sensing images. No study has been published for the 2021 Muli fire, and the area burned in Muli County in 2021 has not been monitored in the remote sensing fire product MOD14A2 resolution due to its large spatial resolution (1 km). Therefore, this method can extract the burning area of smaller fires more accurately, but the extraction accuracy is lower for larger fires such as the 2019 fire, which may be potentially due to fire having heterogeneous effects at scales finer than 30 m, particularly in non-stand replacing fires, resulting in high sub-pixel variability in tree mortality (Schroeter & Foster, 2004; Howe et al., 2022) and frequency of smaller-sized unburned patches. However, the larger the area, the greater the heterogeneity of fire images presented, so it is also a question of whether to choose a more accurate way, such as deep learning, to extract the burning area in the method for a higher spatial resolution. In addition, the accuracy of this method for extracting heavily burned area is higher than that of mildly burned area, which is similar to the study of Zhang et al. (2021) and Silva-Cardoza et al. (2022), In addition, the novelty of this method lies in the rapid acquisition of before and after fire images through the GEE cloud computing platform. GEE and the developed algorithm are very attractive and suitable for providing near real-time burned area maps in that both burned areas and the different levels of burn severity can be identified automatically and without using fixed threshold values. So the method proposed in this article can quickly and accurately extract the burned area and provide important information for the subsequent assessment of ecological changes before and after the fire.

The changes in vegetation cover before and after the fire have been studied (Zhou et al., 2019), but fire also brings changes to the ecological environment, such as soil moisture and heat. So the ecological changes of different degrees of fire also provide basic information for the subsequent environmental restoration. In this article, the ecological environment changes before and after the fires were evaluated using the RSEI remote sensing ecological index, and it is obvious to see in the three fires that the ecological environment changes more in the heavy fires, so whether to intervene artificially in the ecological environment restoration of the heavily burned area is also one of the future research priorities.

In addition, the natural conditions of the study area do create prerequisites for fires to occur, as it is located in the Hengduan Mountains, with complex topography, high mountains and dense forests. Most areas are primitive forests with dense vegetation, where Yunnan pine and other species commonly grow and are rich in oil. Together with the long-term accumulation of dead branches and leaves forming the ground humus layer, all provide combustible material conditions for forest fires to break out. In addition, the mountain landscape is complex, and the terrain is highly undulating; fire spread was accelerated because of the steep mountain environment. The region should focus on highlighting the mechanized forest firefighting professional team equipment construction to improve the ability to deal with forest fires.

Furthermore, what measures should be taken to restore the vegetation in the burned area? It should be considered the fire intensity, fire area and the natural renewal characteristics of the original vegetation in the area. With time, the mildly burned areas will naturally recover to the pre-fire state, and no excessive artificial disturbance measures are usually needed. For heavily burned areas, if the soil conditions are good, can be considered for vegetation restoration measures to speed up forest restoration and achieve economic benefits as early as possible, such as artificial or semi-artificial disturbances. So which stage of fire area restoration to take disturbance measures and what disturbance measures to take are essential elements of scientific research; if the stand conditions of burned areas are poor, such as high mountains and steep slopes, infertile soil, *etc*., such as March 30, 2019, Sichuan Muli County heavy burned area, it is not suitable to take more artificial disturbance measures except for the necessary aircraft seeding to provide seed source.

## Conclusions

Muli County is a national fire risk area. This study clarified the burned area caused by three fires based on the dNBR index calculated from sentinel-2 remote sensing images, while combining the OTSU threshold and the decision tree methods. On March 30, 2019, the burned area was 120.82 hm^2^, of which the heavily burned area (forest loss area) was 43.20 hm^2^ and the mildly burned area (fire traced area) was 77.62 hm^2^; on March 28, 2020, the burned area was 19,254.82 hm^2^, of which 9,574.57 hm^2^ is heavily burned area (forest loss area), and 9,680.25 hm^2^ is mildly burned areas (fire traced area), March 30, 2019, burned area is 226.40 hm^2^, of which 66.81 hm^2^ is heavily burned area (forest loss area) and 159.59 hm^2^ is mildly burned areas (fire traced area). The accuracy verification with the visual interpretation results obtained 97.69%, 89.32%, and 96.37% extraction accuracy based on field survey data. This result improved our understanding of the ecological quality changes through a forest fire and helped the post-fire vegetation recovery.

Meanwhile, the RSEI remote sensing index was used to quantitatively evaluate the ecological environment changes in the three fires. It concluded that RSEI after the three forest fires was significantly lower than that before the fires, indicating that the fires brought different degrees of damage to the ecological environment in the area. Also, the RSEI difference of the heavily burned area fires was higher than that of the mildly burned areas; from the two dimensions of heavily burned areas and mildly burned areas, the area changes of forest RSEI before and after the three fires were quantified by using the transfer matrix. It is quantitatively analyzed the damage caused by fire to the ecosystem.

## Supplemental Information

10.7717/peerj.14557/supp-1 Supplemental Information 1 Burned areas images.

10.7717/peerj.14557/supp-2 Supplemental Information 2 RSEI images.

10.7717/peerj.14557/supp-3 Supplemental Information 3 The code to extract burned areas and calculate RSEI in GEE.

## Additional Information and Declarations

### Data Availability

The following information was supplied regarding data availability: The raw data and code are available in the Supplemental Files.
