# Supplementary figures and images for: Remote sensing technology for rapid extraction of burned areas and ecosystem environmental assessment
Source: PeerJ. 2023 Feb 6;11:e14557. doi: 10.7717/peerj.14557 (PMC9910190; doi:10.7717/peerj.14557)

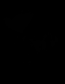

Supplement: Supplemental Information 1 [file peerj-11-14557-s001.zip › Burned areas/Heavy_fire_area_2019.tif]

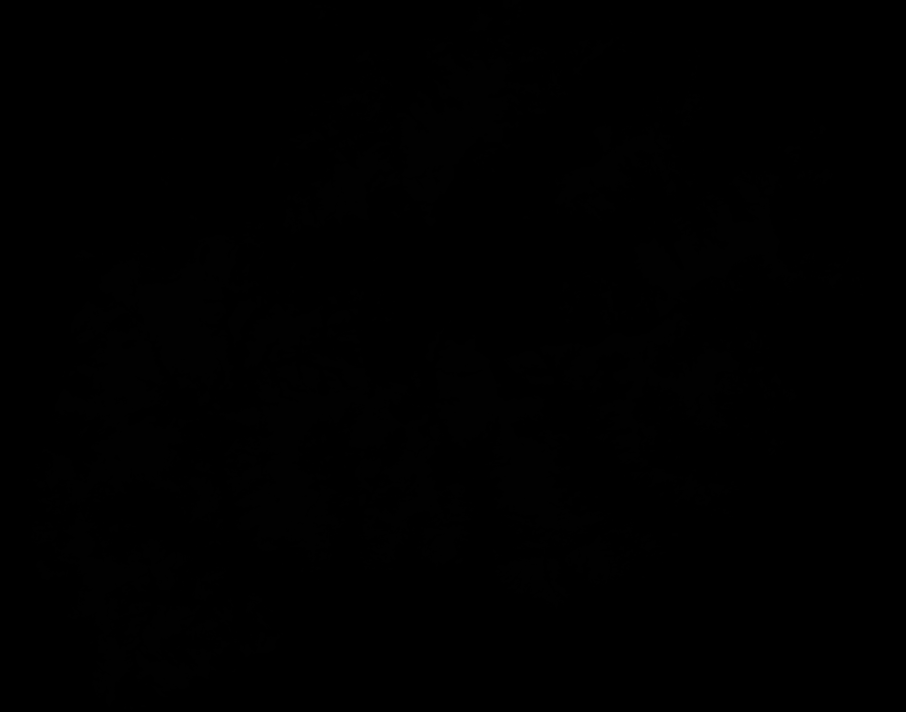

Supplement: Supplemental Information 1 [file peerj-11-14557-s001.zip › Burned areas/Heavy_fire_area_2020.tif]

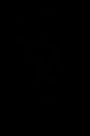

Supplement: Supplemental Information 1 [file peerj-11-14557-s001.zip › Burned areas/Heavy_fire_area_2021.tif]

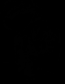

Supplement: Supplemental Information 1 [file peerj-11-14557-s001.zip › Burned areas/Mild_fire_area_2019.tif]

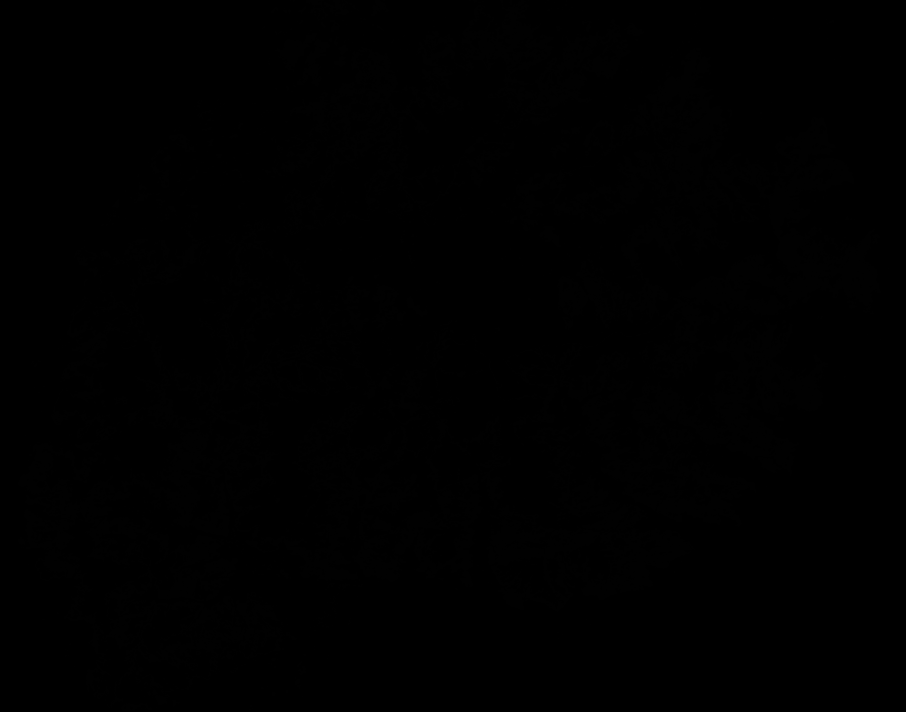

Supplement: Supplemental Information 1 [file peerj-11-14557-s001.zip › Burned areas/Mild_fire_area_2020.tif]

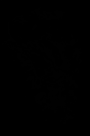

Supplement: Supplemental Information 1 [file peerj-11-14557-s001.zip › Burned areas/Mild_fire_area_2021.tif]
